# Comparison between PET template-based method and MRI-based method for cortical quantification of florbetapir (AV-45) uptake in vivo

**DOI:** 10.1007/s00259-013-2656-8

**Published:** 2013-12-19

**Authors:** L. Saint-Aubert, F. Nemmi, P. Péran, E. J. Barbeau, P. Payoux, F. Chollet, J. Pariente

**Affiliations:** 1 Inserm, imagerie cérébrale et handicaps neurologiques UMR 825, Centre Hospitalier Universitaire de Toulouse, Pavillon Baudot CHU Purpan lace Dr Baylac, 31059 Toulouse, France; 2 Université de Toulouse, UPS, imagerie cérébrale et handicaps neurologiques UMR 825, Centre Hospitalier Universitaire de Toulouse, Toulouse, France; 3 Université de Toulouse, UPS, centre de recherche cerveau et cognition, France, CNRS, CerCo, Toulouse, France; 4 Service de neurologie, pôle neurosciences, Centre Hospitalier Universitaire de Toulouse, Toulouse, France; 5 Service de Médecine Nucléaire, Pôle Imagerie, Centre Hospitalier Universitaire de Toulouse, Toulouse, France

**Keywords:** Alzheimer’s disease, Florbetapir, Amyloid, PET imaging, Cortex

## Abstract

**Purpose:**

Florbetapir (AV-45) has been shown to be a reliable tool for assessing in vivo amyloid load in patients with Alzheimer’s disease from the early stages. However, nonspecific white matter binding has been reported in healthy subjects as well as in patients with Alzheimer’s disease. To avoid this issue, cortical quantification might increase the reliability of AV-45 PET analyses. In this study, we compared two quantification methods for AV-45 binding, a classical method relying on PET template registration (route 1), and a MRI-based method (route 2) for cortical quantification.

**Methods:**

We recruited 22 patients at the prodromal stage of Alzheimer’s disease and 17 matched controls. AV-45 binding was assessed using both methods, and target-to-cerebellum mean global standard uptake values (SUVr) were obtained for each of them, together with SUVr in specific regions of interest. Quantification using the two routes was compared between the clinical groups (intragroup comparison), and between groups for each route (intergroup comparison). Discriminant analysis was performed.

**Results:**

In the intragroup comparison, differences in uptake values were observed between route 1 and route 2 in both groups. In the intergroup comparison, AV-45 uptake was higher in patients than controls in all regions of interest using both methods, but the effect size of this difference was larger using route 2. In the discriminant analysis, route 2 showed a higher specificity (94.1 % versus 70.6 %), despite a lower sensitivity (77.3 % versus 86.4 %), and D-prime values were higher for route 2.

**Conclusion:**

These findings suggest that, although both quantification methods enabled patients at early stages of Alzheimer’s disease to be well discriminated from controls, PET template-based quantification seems adequate for clinical use, while the MRI-based cortical quantification method led to greater intergroup differences and may be more suitable for use in current clinical research.

**Electronic supplementary material:**

The online version of this article (doi:10.1007/s00259-013-2656-8) contains supplementary material, which is available to authorized users.

## Introduction

Alzheimer’s disease (AD) is a major current public health issue and has been the target of numerous clinical studies in recent decades to improve its diagnosis from the earliest stages, and to develop new therapies to slow its progression. The diagnosis of probable AD relies on clinical criteria that are constantly being updated [1, 2]. Early diagnosis of AD, in particular at the prodromal stage – when the first symptoms appear – is now also possible in clinical research thanks to established research criteria based on multimodal assessments. These include amyloid pathology assessment using various methods, such as cerebrospinal fluid (CSF) sampling or amyloid-specific imaging by PET [2, 3]. Several amyloid ligands have been studied in vivo, two of which are widely used in current clinical research: [^11^C-]PiB [4] and [^18^ F-]Florbetapir (AV-45) [5]. Both have now been proven to be reliable tools for assessing the amyloid burden in the brain of patients with AD compared to healthy controls [6–11]. Our group has recently published a study on AV-45 binding in patients with prodromal AD [12], and a case report showing amyloid pathology using AV-45 PET imaging during presymptomatic stages of AD [13].

As the present research criteria include amyloid biomarkers, and now that the Food and Drug Administration [14] and the European Medicines Agency (http://www.ema.europa.eu/) have approved the use of AV-45 imaging for clinical purposes [15], it is important to seek out the most reliable method for analysing AV-45 imaging. Most research studies have investigated AV-45 uptake using quantification methods based on target-to-cerebellum standard uptake values (SUVr) [5, 8, 16]. Nonspecific AV-45 binding has been reported in white matter [9]. This nonspecific binding may affect AV-45 quantification when using whole-brain analyses. To avoid this issue, a few studies have started to focus on AV-45 binding in grey matter only [17, 18]. In their study, Rodrigue et al. excluded white matter from their analyses by using AV-45 images from young healthy subjects as masks assuming that no specific binding would be found in a young healthy population [18]. In another recent study, La Joie et al. applied a grey matter mask to AV-45 images that were normalized in MNI space [17].

In the present study, we compared two methods for AV-45 cortical quantification, the first one quantifying regional mean SUVr measured using an AV-45 template, and the second an optimized method based on cortical signal extraction of SUVr using individually segmented T1 MRI sequences.

## Materials and methods

### Participants

All participants gave their written informed consent. The study was approved by the local ethics committee (Comité de Protection des Personnes Sud-Ouest et Outre-Mer I) and the French Agency for the Safety and Security of Medical Devices (Agence Française de Sécurité Sanitaire des Produits de Santé, reference A90605-58).

For this study, patients over 65 years old and with AD at the prodromal stage [3] were recruited. They were all recruited from the outpatient memory clinic (Neurology Department, University Hospital, Toulouse, France). Matched cognitively normal (CN) subjects were recruited from among patients’ relatives or using recruitment posting in public places. All participants underwent a neuropsychological and a medical assessment, brain MRI, FDG PET and AV-45 PET imaging. CSF sampling was also performed in patients only. To be included in the study, patients had to show preserved autonomy in daily life (Clinical Dementia Rating ≤0.5) but a memory complaint of more than 6 months duration attested to by isolated memory impairment on neuropsychological assessment [19] and one or more of the following features:

- Medial temporal lobe atrophy assessed on MRI scan (sequences detailed below)
- Temporoparietal hypometabolism pattern on cerebral FDG PET scan (classical clinical acquisitions)
- Abnormal CSF biomarkers according to published criteria [20, 21]

Patients were not included if they had a concomitant neurological or psychiatric disease, or if they were affected by any clinically significant pathology that could explain the memory complaint. Significant white matter hyperintensities found on T2-weighted MRI images were a reason for exclusion.

CN subjects were included if they had no memory complaint, no neurological or psychiatric disease history, and if they had no first-degree relatives with AD. They were excluded if they showed significant white matter hyperintensities on T2-weighted MR images or any cognitive impairment on neuropsychological assessment.

More details on this population recruitment have been published elsewhere [12].

### MRI and AV-45 acquisitions

#### Brain MRI scans

MRI scans were performed in all participants using a 3-T imager (Intera Achieva; Philips, Best, The Netherlands). High-resolution anatomical images using a 3-D T1-weighted sequence (in-plane resolution 1 × 1 mm, slice thickness 1 mm, repetition time/echo time/inversion time 8,189/3.75/1,012.2 ms, flip angle 8°, field of view 240 × 256, and 160 contiguous slices) and a T2-weighted sequence (reconstructed resolution 0.45 × 0.45 × 3 mm, repetition time/echo time 4,132/80 ms, flip angle 90°, field of view 240 × 184, and 43 slices) were obtained. CN subjects underwent the same MRI acquisitions as patients.

#### AV-45 PET scans

All participants underwent a PET scan using AV-45 amyloid marker. All scans were performed on the same hybrid PET/CT scanner (Biograph 6 TruePoint Hirez; Siemens Medical Solutions) using 3-D detection mode producing images with voxels of 1 × 1 × 1.5 mm and a spatial resolution of approximately 5 mm full-width at half-maximum at the centre of the field of view). Both CT and PET scans were acquired. Cerebral emission scans were started 50 min after injection of 3.7 MBq/kg weight of AV-45 and lasted for 20 min. PET data were corrected for partial volume effects using the point spread function (PSF) modelling implemented by Siemens (HD-PET©). Briefly the manufacturer previously measured the response of a point source from many points in its FOV and incorporated measured PSFs in the reconstruction algorithms. Using measured PSFs, HD-PET effectively positions the lines of response in their actual geometric location, which reduces blurring and distortion in the final images.

Three independent raters (two nuclear medicine physicians and one radiologist physicist) with extensive experience reading AV-45 PET scans, blind to clinical information, examined all PET scans. A two-point rating scale (0 normal, 1 amyloid-positive) was used. AV-45 PET scans were considered amyloid-positive when the rating score was 1 for at least two raters. The maximum delay between the two imaging examinations was 3 months.

### Image processing

#### Route 1: Template-based AV-45 quantification

AV-45-PET scans were linearly registered with 12 degrees of freedom (DOF) and trilinear interpolation onto a template from Avid (http://www.avidrp.com/), which is in the Montreal Neurological Institute (MNI) space, using FLIRT (FSL library tools) [22].

**Fig. 1:**
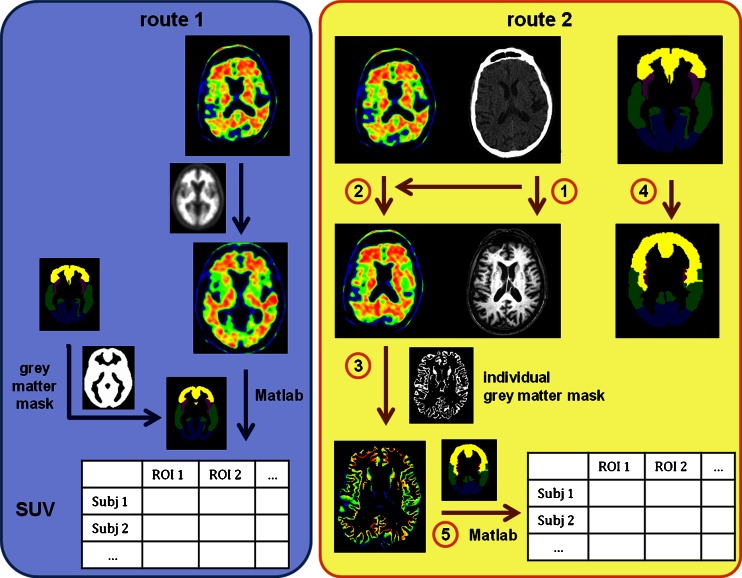
AV-45 quantification procedures: **a** cortical PET template-based (*route 1*) and **b** cortical MRI-based (*route 2*). **a** In route 1, AV-45 acquisitions were linearly registered onto a template from Avid (http://www.avidrp.com/), and regional uptake was quantified for each subject in the MNI space using the AAL atlas [24] that was masked with a grey matter probability map. Uptake values were collected using Matlab. **b** In route 2, for each subject, the CT scan acquired together with the AV-45 image was registered onto the MRI anatomical space defined by the T1 image of the subject concerned (*1*). The transformation matrix obtained was then applied to the AV-45 image of the subject so that the AV-45 image was in the T1 space (*2*). A binarized grey matter mask obtained from MRI segmentation was applied to the transformed AV-45 image (*3*). The AAL atlas was also registered onto each individual T1 space using the inverse of the transformation matrix from the T1 registration onto MNI space (*4*). Regional cortical uptake was collected for each subject using Matlab (*5*). *Arrows* represent transformation (*Subj* subject)

Regional cortical AV-45 mean SUV was measured in each subject using a Matlab (The MathWorks®) script developed in-house (Fig. 1a). Mean global SUVs, SUVs in the five lobes, and SUVs in specific regions of interest (ROIs: orbitofrontal, anterior cingulate, posterior cingulate and precuneus) were calculated. These regions were selected as they have shown different AV-45 binding in studies comparing AD patients to CN subjects [8, 9, 12, 16, 23]. These regions were defined using the anatomical automatic labelling (AAL) atlas [24] provided by MRIcron software, that was linearly registered (12 DOF, trilinear interpolation) onto the AV-45 template and binarized. To minimize the bias due to nonspecific white matter binding, the AAL atlas was masked for grey matter. To do so, we used the grey matter probability map from SPM8 (Wellcome Trust Centre for Neuroimaging, London, UK), that was binarized using a threshold of 0.3 – meaning that only voxels with a probability greater than 30 % of being grey matter were selected.

SUVs were then normalized (SUVr) to the whole cerebellar mean uptake (vermis excluded) using Matlab and pooled by group for statistical comparison (Fig. 1a).

#### Route 2: Cortical MRI-based AV-45 quantification

Grey matter was segmented from the 3-D T1 anatomical image of each participant using FAST and FIRST (FSL library tools), and the resulting grey matter mask was binarized using a threshold of 0.3 – as used for route 1 [25]. Then, for each subject, the CT scan obtained during PET data acquisition was linearly registered (12 DOF) onto the relative T1 anatomical image. The transformation matrix obtained was applied to the AV-45 image of the subject so that the AV-45 image was in the T1 space. The grey matter mask from T1 segmentation (see above) was applied to the transformed AV-45 image. The AAL atlas [24] was also registered from MNI space to each individual T1 space. To do this, the linear transformation matrix from the registration of each single subject’s T1 onto MNI space was first calculated. The inverse of this matrix was then applied for each subject to the AAL atlas to obtain ROIs in the subject’s space. Finally, the registered atlases were binarized.

The cerebral regions studied were the same as those studied in route 1. Values of regional cortical AV-45 mean SUV were obtained for each subject using the same Matlab script as in route 1 (Fig. 1b). SUVs were normalized (SUVr) to the whole cerebellar mean uptake (vermis excluded, identical definition to that used in route 1) and pooled by group for statistical comparison.

### Statistical analyses

The threshold for significance was set at *p* < .05. For the neuropsychological assessments, groups were compared using the Mann-Whitney or chi-squared statistical tests. A parametric paired *t* test with Bonferroni-Holmes correction for multiple comparisons was used to compare SUVr values between the two routes for both groups and to compare regional uptake differences between groups for both quantification methods. The effect sizes were assessed using Cohen’s *d* [26]. Standard deviations were weighted by sample size as the number of participants was not equal between groups [27].

 For discriminant analysis of the imaging data from both methods, receiver operating characteristics analyses were performed on the mean global SUVr values to study the diagnostic power. The areas under the curve (AUC) were calculated and sensitivity and specificity were computed at the optimal cut-off points. D-prime values as well as their associated bias measures C were also computed for several cut-off values: the value leading to the same sensitivity and specificity for both routes, the value leading to the best sensitivity and specificity trade-off for route 1, and the value leading to the best sensitivity and specificity trade-off for route 2.

## Results

Twenty-two patients with prodromal AD and 17 CN subjects were included in the study (see supplementary data 1 for individual profiles on research criteria). The two groups were matched for age and level of education (see Table 1 for clinical and neuropsychological data).

**Table 1:** Clinical and neuropsychological assessment

	Prodromal patients (*n* = 22)	CN subjects (*n* = 17)	*p* value
Age (years)	72.4 ± 5.0	69.9 ± 4.8	.110
Gender (M/F)	12/10	7/10	.408
Level of education (years)	11.3 ± 2.7	12.8 ± 3.3	.163
Disease duration (years)	3.8 ± 3.6	–	–
Daily-life autonomy (CDR scale)	0.5 ± .0	0.0 ± .0	.001*
Global cognitive state (MMSE)	25.7 ± 1.4	28.4 ± .7	<.001*
Anterograde verbal memory (FCSRT test)				
Sum of free recalls (/48)	11.6 ± 5.9	32.2 ± 4.6	.001*
Sum of free and cued recalls (/48)	28.7 ± 11.9	46.6 ± 1.9	.001*

*CDR* Clinical Dementia Rating, *FCSRT* Free and Cued Selective Recall Reminding test

*p < .05

Visual assessment of AV-45 PET scans revealed amyloid-positive profiles in 18 patients out of 22, and in 2 CN subjects out of 17.

### Comparison of SUVr values between the two routes for both groups

In the patient group, regional SUVr values in the insula and the orbitofrontal region were significantly lower using route 1 than route 2, while SUVr values in the posterior cingulate were significantly higher using route 1. In the CN group, mean global as well as frontal, parietal and posterior cingulate SUVr values were significantly higher using route 1 than route 2 (Table 2).

**Table 2:** Comparison between the two quantification methods for both groups. AV-45 mean global and target-to-cerebellum SUVr values and associated standard deviations are shown for the two groups using PET template-based quantification (route 1) and MRI-based quantification (route 2). Significant *p* values (<.05) are shown

Region	Patients			CN subjects		
	Route 1	Route 2	*p* value	Route 1	Route 2	*p* value
Global	1.47 ± .24	1.50 ± .32	–	1.25 ± .13	1.18 ± .09	.010
Frontal lobes	1.49 ± .28	1.54 ± .38	–	1.29 ± .17	1.19 ± .13	.010
Temporal lobes	1.42 ± .22	1.45 ± .30	–	1.19 ± .13	1.16 ± .07	–
Insular lobes	1.37 ± .27	1.46 ± .32	.045	1.14 ± .13	1.19 ± .11	–
Parietal lobes	1.54 ± .25	1.51 ± .35	–	1.26 ± .14	1.15 ± .09	.002
Occipital lobes	1.43 ± .22	1.49 ± .31	–	1.24 ± .13	1.22 ± .08	–
Orbitofrontal	1.47 ± .31	1.60 ± .41	.011	1.22 ± .17	1.23 ± .16	–
Anterior cingulate	1.59 ± .31	1.67 ± .41	–	1.36 ± .20	1.30 ± .16	–
Posterior cingulate	1.82 ± .31	1.74 ± .36	.021	1.55 ± .27	1.36 ± .14	.005
Precuneus	1.56 ± .30	1.56 ± .36	–	1.20 ± .14	1.14 ± .09	–

### Imaging comparison between groups

Both methods showed significantly higher AV-45 uptake in the patient group than in the CN subjects in all ROIs.

#### Route 1 (AV-45 template-based quantification)

**Fig. 2:**
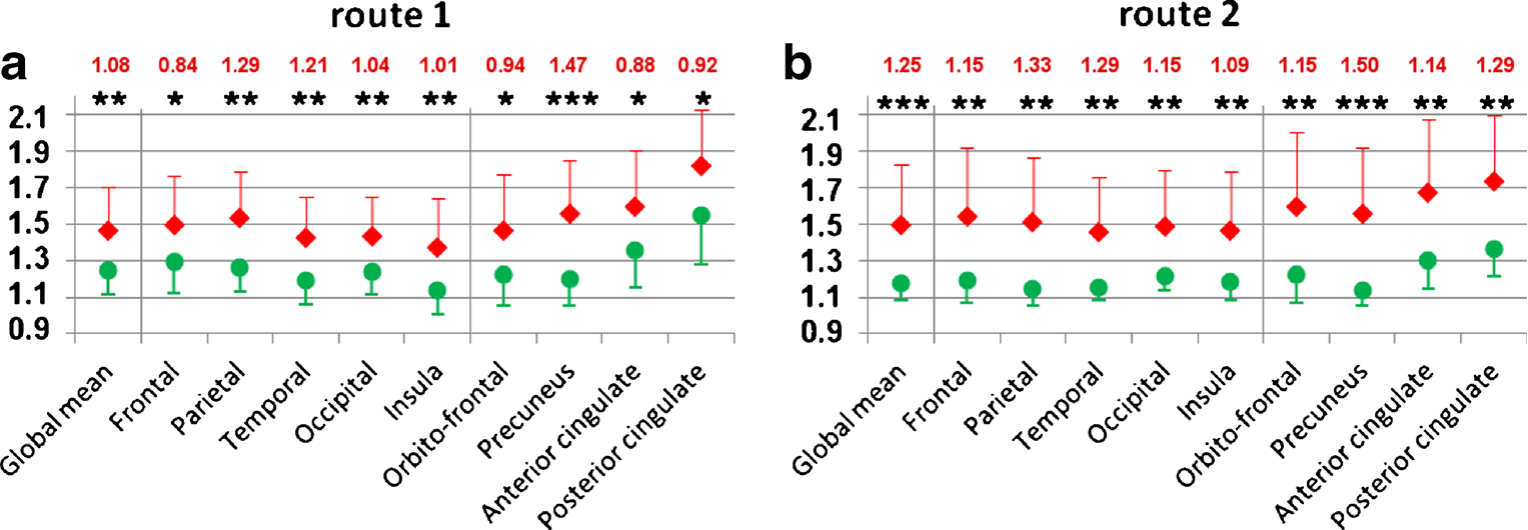
Target-to-cerebellum cortical AV-45 uptake ratios using **a** route 1 (the PET template-based method) and **b** route 2 (the MRI-based method). Mean values in all ROIs are shown with associated standard deviations (*red diamonds* patients, *green circles* control subjects) *p < .05, **p < .01, ***p < .001. Cohen’s *d* values for each region are given in *red characters*

Compared to the CN subjects, the patients showed a significantly higher uptake of AV-45 in the whole brain (*p* = .002), in every cerebral lobe (frontal *p* = .013, parietal *p* = .001, temporal *p* = .003, insula *p* = .007, occipital *p* = .008) and each ROI (orbitofrontal *p* = .018, anterior cingulate *p* = .010, posterior cingulate *p* = .014, precuneus *p* < .001; Fig. 2a).

#### Route 2 (MRI-based AV-45 quantification)

Compared to the CN group, the patients group showed a significantly higher uptake of AV-45 in the whole cortex (*p* < .001), in every cerebral lobe (frontal *p* = .002, parietal *p* = .001, temporal *p* = .001, insula *p* = .002, occipital *p* = .003), and each ROI (orbitofrontal *p* = .002, anterior cingulate *p* = .001, posterior cingulate *p* = .001, precuneus *p* < .001; Fig. 2b).

#### Effect size

AV-45 uptake was higher in patients than in CN subjects with both methods. Cohen’s *d* values were calculated for all regions. They were always higher with route 2 than with route 1 (Fig. 2).

### Discriminant analysis

**Fig. 3:**
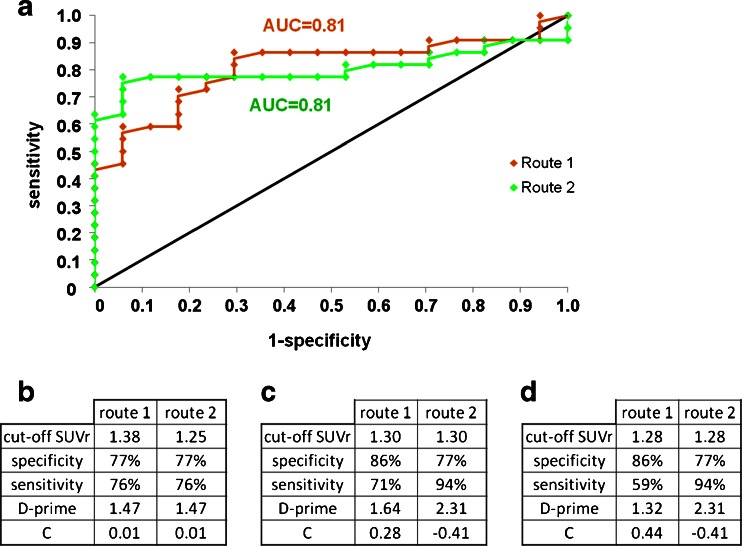
Receiver operating characteristics curves for route 1 and route 2 (**a** *orange curve* route 1, *green curve* route 2). **b–d** D-prime values and the corresponding values of the bias measure C for three different SUVr values: **b** cut-off value for same sensitivity/specificity for route 1 and route 2, **c** cut-off value for best sensitivity/specificity for route 1, **d** cut-off value for best sensitivity/specificity for route 2

Discriminant analysis of the mean global AV-45 SUVr values from route 1 showed the highest sensitivity (86.4 %) and specificity (70.6 %) with a cut-off value of 1.67 (AUC 0.81). For this cut-off value, D-prime was 1.64 for route 1 and 2.31 for route 2 (Fig. 3).

In contrast, discriminant analysis of the mean global AV-45 SUVr values from route 2 showed the highest sensitivity (77.3 %) and specificity (94.1 %) with a cut-off value of 1.28 (AUC 0.81). For this cut-off value, D-prime was 1.32 for route 1 and 2.31 for route 2 (Fig. 3).

## Discussion

In this study, we compared two quantitative methods for assessing cortical AV-45 target-to-cerebellum uptake in a population of patients with prodromal AD matched to CN subjects. Route 1 relied on cortical quantification using a standardized PET template. Route 2 relied on cortical uptake using cortical signal extraction from individual MRI sequences. We showed that (1) there were significant differences between the SUVr values from route 1 and route 2 in the patients and CN groups; (2) global quantification relying on the cortex only (route 2) showed a greater difference between SUVr of prodromal patients and SUVr of CN subjects than the template-based registration (route 1). To our knowledge, this is the first study showing significant differences between PET template-based and MRI-based cortical AV-45 quantification methods.

Patients were included if they were diagnosed with prodromal AD according to research criteria [3]. Amyloid burden is thought to appear early, before the first clinical symptoms, and to have reached a high level at the prodromal stage. In this study, both methods showed significantly higher AV-45 uptake in prodromal patients than in matched CN subjects, both global uptake and uptake in all neocortical regions.

To avoid interference from white matter uptake in our quantification methods, we used grey matter masks. However, the same mask was used for all PET images in route 1, all being in MNI space, while a specific grey matter mask was used for each participant derived from segmentation of the subject’s own MRI scan in route 2, potentially leading to more precision in cortical quantification. This may be the reason for significant differences observed between the two routes. In the CN group there were significant decreases in global uptake and in frontal, parietal and posterior cingulate uptakes using route 2 compared to route 1. It is known that CN subjects show important nonspecific AV-45 binding in white matter, which may affect whole-brain quantification. Our PET template-based quantification method (route 1) may have only partially suppressed the effect of white matter uptake, while the individual MRI-based method (route 2) produced lower cortical SUVr values in CN subjects. Similar results were found for the posterior cingulate region in the patient group, with significantly lower uptake found using route 2. One hypothesis that may be proposed is that the white matter PET signal could easily spill over onto the posterior cingulate because of the latter’s proximity to dense white matter fibres. As in the CN subjects, the MRI-based method would minimize the effect of white matter uptake in this region in patients. We also found significant increases in the uptake values in the insula and the orbitofrontal regions of patients when using route 2. This may be due to high specific amyloid binding that would be reduced by some white matter signal “surviving” in route 1. Further studies testing such hypotheses are necessary.

We used MRI for individual grey matter segmentation since classical T1-weighted MRI sequences have higher resolution than PET images and since most patients nowadays undergo clinical MRI scans for diagnostic purposes. Many studies on amyloid imaging have used visual assessment to determine the uptake profiles of amyloid markers and to classify subjects as “positive” or “negative”. Although it may be the fastest way so far found to assess AV-45 uptake profiles in clinical routine, visual assessment has shown some limitations [28] and should be considered with caution. The use of SUV quantification, and in particular cortical quantification, should provide more precise results. It is thus crucial to develop fast, reproducible techniques.

A recent study investigated similar questions regarding analyses of amyloid PET imaging using PiB in AD patients and CN subjects [25]. The authors compared a method using a PiB template for normalization, and one method using MRI for grey matter segmentation and normalization. Both methods included transformation into MNI space – which was not the case in our study. The authors showed that the PET template-based and MRI-based methods were slightly different, with systematically higher, but comparable, values for the PET template-based method in every group. In our study, the results of our two methods were not comparable. Route 2 (MRI-based method) showed greater differences and a larger effect sizes of the cortical SUVr values of prodromal AD patients and CN subjects than route 1. However, we used a different approach to that of Edison et al. regarding the MRI-based method, as we performed our analyses in the individual space in all subjects to stay closest to the individual grey matter anatomy. Such MRI-based cortical quantification would thus lead to greater accuracy in individual regional quantification. Besides, our study used the amyloid biomarker AV-45, which has shown higher nonspecific binding than PiB [16]. This could also partly explain the significant differences we found between route 1 and route 2 that were not found by Edison et al. [25]. In another recent study, Landau et al. investigated amyloid ligand uptake using PiB and AV-45 in a patients with mild cognitive impairment compared to healthy subjects [29]. They used similar methods to our routes 1 and 2 for cortical quantification, and found similar results between the two routes. These results, at odds with ours, might be accounted for by differences in the size of the selected ROIs in the two studies.

On discriminant analysis, the two routes showed different patterns. Route 1 showed a higher sensitivity than route 2 (86.4 % versus 77.3 %, respectively), but a lower specificity (70.6 % versus 94.1 %, respectively). The high specificity of route 2 corresponds to only one false-positive result, which is in accordance with results of other studies [10, 30, 31]. This means that a cortical MRI-based quantification gives fewer false-positive results among CN subjects, and may be more suitable for use in clinical research. On the other hand, the conventional AV-45 template-based method gave fewer false-negative results among patients. As a consequence, such a method appears adequate for clinical use if the aim is to confirm the diagnosis in patients presenting with other markers suggestive of AD. The D-prime calculation showed higher values for route 2, suggesting an overall better quality of prediction using MRI-based quantification. Bias measure C confirmed the tendency of route 1 to be sensitive, as opposed to the tendency of route 2 to be specific. Of note, visual assessment showed discrimination results in between, with a sensitivity of 81.8 % and a specificity of 88.2 %.

### Conclusion

This study demonstrated that both cortical MRI-based and PET template-based AV-45 quantifications efficiently discriminate between prodromal patients and healthy subjects. Clinical diagnosis may rely on PET template-based quantification as it discriminates better between patients and healthy subjects and it is faster and cheaper to perform than MRI-based quantification. On the other hand the latter would be more suitable for research purposes, as only 1 out of 17 CN subjects was misclassified. A further AV-45 quantification study including a larger sample should be performed.

## Electronic supplementary material

Below is the link to the electronic supplementary material.

ESM (DOCX 14 kb)
