# Supplementary material for: Comparison between PET template-based method and MRI-based method for cortical quantification of florbetapir (AV-45) uptake in vivo
Source: Eur J Nucl Med Mol Imaging. 2013 Dec 19;41(5):836–43. doi: 10.1007/s00259-013-2656-8 (PMC3978219; doi:10.1007/s00259-013-2656-8)
Supplement: Supplementary file 1 — ESM (DOCX 14 kb) [file 259_2013_2656_MOESM1_ESM.docx]

|  | Memory impairment | Atrophy | Hypometabolism | CSF suggestive of AD | amyloid-profile on AV-45 visual assessment |
| --- | --- | --- | --- | --- | --- |
| Patient 1 | + | - | + | + | + |
| Patient 2 | + | + | + | + | + |
| Patient 3 | + | + | - | + | + |
| Patient 4 | + | + | + | + | + |
| Patient 5 | + | - | + | + | - |
| Patient 6 | + | + | + | + | + |
| Patient 7 | + | + | + | + | + |
| Patient 8 | + | + | + | + | + |
| Patient 9 | + | + | + | + | - |
| Patient 10 | + | + | + | NA | - |
| Patient 11 | + | + | + | + | + |
| Patient 12 | + | - | + | NA | + |
| Patient 13 | + | + | - | + | + |
| Patient 14 | + | - | + | **-** | + |
| Patient 15 | + | + | + | NA | + |
| Patient 16 | + | + | + | + | + |
| Patient 17 | + | + | + | + | + |
| Patient 18 | + | + | - | + | + |
| Patient 19 | + | + | + | + | + |
| Patient 20 | + | + | + | + | + |
| Patient 21 | + | - | - | + | - |
| Patient 22 | + | + | + | + | + |

**Supplementary data 1. Individual profiles of patients on criteria from Dubois et al. 2007 [3]**

Fulfillment of a criteria is indicated with “+”. Absence of fulfillment is indicated with “-”. CSF=cerebrospinal fluid. Visual assessment score on AV-45 PET scan is also indicated (“+” accounting for amyloid-positive profile, and “-” for amyloid-negative profile). NA=not available.
